# Weight Trimming and Propensity Score Weighting

**DOI:** 10.1371/journal.pone.0018174

**Published:** 2011-03-31

**Authors:** Brian K. Lee, Justin Lessler, Elizabeth A. Stuart

**Affiliations:** 1 Department of Epidemiology and Biostatistics, Drexel University School of Public Health, Philadelphia, Pennsylvania, United States of America; 2 Department of Epidemiology, Johns Hopkins Bloomberg School of Public Health, Baltimore, Maryland, United States of America; 3 Department of Mental Health, Johns Hopkins Bloomberg School of Public Health, Baltimore, Maryland, United States of America; 4 Department of Biostatistics, Johns Hopkins Bloomberg School of Public Health, Baltimore, Maryland, United States of America; University of Modena and Reggio Emilia, Italy

## Abstract

Propensity score weighting is sensitive to model misspecification and outlying weights that can unduly influence results. The authors investigated whether trimming large weights downward can improve the performance of propensity score weighting and whether the benefits of trimming differ by propensity score estimation method. In a simulation study, the authors examined the performance of weight trimming following logistic regression, classification and regression trees (CART), boosted CART, and random forests to estimate propensity score weights. Results indicate that although misspecified logistic regression propensity score models yield increased bias and standard errors, weight trimming following logistic regression can improve the accuracy and precision of final parameter estimates. In contrast, weight trimming did not improve the performance of boosted CART and random forests. The performance of boosted CART and random forests without weight trimming was similar to the best performance obtainable by weight trimmed logistic regression estimated propensity scores. While trimming may be used to optimize propensity score weights estimated using logistic regression, the optimal level of trimming is difficult to determine. These results indicate that although trimming can improve inferences in some settings, in order to consistently improve the performance of propensity score weighting, analysts should focus on the procedures leading to the generation of weights (i.e., proper specification of the propensity score model) rather than relying on ad-hoc methods such as weight trimming.

## Introduction

Propensity score methods are a means of controlling for confounding in non-experimental studies [1]. Briefly, the propensity score is the probability of receiving a treatment conditional on observed covariates. By conditioning on the propensity score one can achieve an unbiased estimate of the treatment effect, assuming no unmeasured confounding. Conditioning on the propensity score typically occurs through weighting, matching, stratification, or regression adjustment. Although any of these methods can be used for propensity score adjustment, some evidence suggests that weighting and matching may be optimal in some instances [2]. For example, in studies involving complex sampling methods where units have differential probabilities of inclusion, propensity score weighting may be particularly recommended [3].

Propensity score weighting is similar with survey sampling weighting, which accounts for over- or under- sampling by weighting the sample to represent the population from which the sample was drawn. In the propensity score context, weighting is used to account for different probabilities of exposure between comparison groups. Different weighting schemes are possible. The most frequently used is inverse probability of treatment weighting, where exposed and unexposed individuals are weighted to represent the population. A variation we use here, which is described in detail below, weights the unexposed group to resemble the exposed group. Propensity score weighting is frequently used in a variety of epidemiological settings to estimate causal effects (e.g. [3], [4], [5], [6], [7], [8]). Diagnostics are a crucial element of using propensity score methods in general, and in particular the key diagnostics are generally those that compare the covariate distributions in the propensity-score-adjusted samples (e.g., the weighted or matched samples), ensuring that the groups are comparable with respect to the observed covariates (see Stuart, 2010 [9], or Rubin, 2001 [10], for further discussion of propensity score diagnostics). A particular diagnostic concern with regard to propensity score weighting is that observations with extremely large weights may unduly influence results and yield estimates with high variance [10], [11], [12]. Because weights are derived directly from propensity scores, misspecified propensity score models are one potential cause for extreme weights. Two possible solutions for extreme propensity score weights due to model misspecification are to improve the specification of propensity score models, and to reduce the impact of extreme weights through trimming [13], [14]. Weight trimming, sometimes referred to as truncation [15], refers to the reduction of weights larger than some value *w_0_* to *w_0_* [16]. In some cases, authors have trimmed low weights smaller than some value *w_0_* to *w_0_*, although we do not consider that method here [15]. Although common in the survey sampling world, weight trimming has not been investigated as thoroughly in propensity score settings.

Machine learning refers to a diverse set of automated classification and prediction algorithms that are commonly used in data mining and artificial intelligence. Several authors have suggested the use of such techniques in propensity score estimation [17], [18], [19], [20], [21], [22] and empirical evidence indicates that these methods can perform well in a variety of scenarios [20], [23]. In a previous study of propensity score estimation using classification and regression tree (CART) methods, we found that certain machine learning data fitting methods could provide substantially better bias reduction and confidence interval coverage compared with logistic regression [24]. In particular, the machine learning methods of boosted CART [19] and random forests [25] provided consistently superior performance. In this manuscript, we build on our previous work and consider the problem of variability and potential outlier status of propensity score weights. In particular we apply weight trimming techniques to determine how trimming influences treatment effect estimates, and whether the effects of trimming vary when propensity scores are estimated using logistic regression versus machine learning methods.

## Methods

### Simulation setup

We used a simulation framework introduced by Setoguchi and colleagues based on real-world claims data modeling statin use [20]. This established simulation setup allows us to investigate weight trimming in scenarios that were explored to answer other questions, therefore ensuring comparability with previously published results. Each simulated dataset consisted of N = 500 observations with a binary exposure, continuous outcome, and 10 covariates (4 associated with both exposure and outcome, 3 associated only with the exposure, and 3 associated only with the outcome). Covariates were generated as standard normal random variables with zero mean and unit variance, and several of the covariates were correlated. The exposure probability at the average of covariates was approximately 0.5. The continuous outcome was generated from a linear combination of the exposure and covariates such that the true effect of exposure equaled −0.4. One thousand datasets were simulated for each of three different scenarios where the true propensity score model had the following properties:

- Scenario 1: additivity and linearity (main effects only)
- Scenario 2: mild non-additivity and non-linearity (three two-way interaction terms and one quadratic term)
- Scenario 3: moderate non-additivity and non-linearity (ten two-way interaction terms and three quadratic terms).

Scenarios 1, 2, and 3 correspond with Scenarios A, E, and G in the study by Setoguchi et al. [20] and our previous study [26]; the formulae used to generate these scenarios are listed in the appendix of their manuscript. In addition, for reproducibility, and to see the details of the simulation settings, R code and all parameter values to generate the simulation datasets are included in Supporting Information Text S1.

### Propensity score estimation

We used R 2.9.2 to estimate propensity scores using the following methods:

- Logistic regression: standard logistic regression estimating probability of treatment from all 10 covariates, with a main effect term for each covariate (no non-linear terms or interactions)
- CART: recursive partitioning; implemented with the *rpart* package with default settings [27]
- Random forests: CART iteratively fitted to repeated samples of the original dataset using random predictors; implemented with the *randomForest* package with default settings [28]
- Boosted CART: iteratively fitted CART to random subsets of data where each new iteration provides greater priority to incorrectly classified observations in the previous tree; implemented using the *twang* package [29] with recommended parameters and an iteration stopping point minimizing the mean of the Kolmogorov-Smirnov test statistics.

For all methods we used the default settings since that is often how these methods are implemented in practice, even if fine-tuning the settings may lead to improved performance for any particular dataset.

### Propensity score weights

Although various weighting schemes have been used with propensity score weights, we choose to perform weighting by the odds to estimate the average treatment effect among the treated. This estimand, which is often of interest in observational studies, refers to the average treatment effect in a population with a covariate distribution similar to that of the sample that received the treatment [3], [8], [19]. Subjects in the treated/exposed group receive a weight of 1, and those in the untreated/unexposed group receive a weight of *p_i_/(1-p_i_)*, where *p_i_* refers to an individual's probability of receiving the treatment (i.e., the individual's propensity score). This weights the control group to resemble the treatment group. In other words, untreated/unexposed subjects who are dissimilar to the exposed/treated group will have a *p_i_* near zero and a weight near zero; untreated/unexposed subjects that are more similar to the exposed/treated group will have a larger *p_i_* and therefore larger weights. The propensity score weights are then incorporated as weights into a standard outcome linear regression model with only the treatment as a predictor variable and no covariates [12]. To better isolate the effects of weight trimming, we do not perform ‘doubly robust’ regression adjustment for covariates after weighting is applied [30].

Trimming was performed using percentile cutpoints [15]. In particular, we trimmed high weights downwards, with cutpoints ranging from the 99^th^ to the 50^th^ percentiles, at 1% intervals. For example, when trimming at the 90^th^ percentile, all weights with value above the 90^th^ percentile were set equal to the 90^th^ percentile. We evaluate the performance of weight trimming by examining the bias (the absolute percentage difference from the true treatment effect), 95% confidence interval (CI) coverage, and standard error of effect estimates.

## Results

Before trimming, propensity score weights for the unexposed group differed by estimation method and scenario (**Table 1**). For all estimation methods, the most complex scenario (3 - moderate non-additivity and non-linearity) increased the proportion of extreme weights. Compared with the other estimation methods, boosted CART produced fewer extreme weights across all scenarios. For example, in scenario 3, the average sum of unexposed observation weights above the 95^th^ percentile (in other words, the average sum of weights for the top 5% of unexposed persons, roughly 12 observations) was as follows for each method: logistic regression  = 82.3; CART  = 59.6; random forests  = 80.6; boosted CART  = 37.1. Spearman correlations of the weights by estimation method are described in **Table 2**. Logistic regression weights were strongly correlated with random forests and boosted CART weights in scenarios 1 and 2 (r from 0.75 to 0.83) but these correlations weakened in scenario 3 (r = 0.63 and 0.65, respectively). CART weights were less strongly correlated with the weights from other methods, with correlations ranging from 0.39 to 0.59. Boosted CART and random forests weights were the most highly correlated, at approximately 0.90 in all scenarios.

**Table 1:** Distribution of Propensity Score Weights for the Unexposed Group by Estimation Method and True Propensity Score Model Scenario.

	1st quartile	Median	3rd quartile	Maximum	Proportion ≥10	Proportion ≥20
**Scenario 1: additivity and linearity**						
Logistic regression	0.30	0.60	1.22	119.5	0.37%	0.05%
CART	0.26	0.37	1.39	49.0	0.16%	0.009%
Random forests	0.41	0.74	1.35	91.5	0.25%	0.04%
Boosted CART	0.21	0.40	0.75	14.3	0.004%	0.000%
**Scenario 2: mild non-additivity and non-linearity**						
Logistic regression	0.21	0.46	1.00	110.1	0.42%	0.07%
CART	0.21	0.31	0.52	37.0	0.13%	0.008%
Random forests	0.31	0.59	1.13	59.7	0.15%	0.02%
Boosted CART	0.16	0.31	0.61	15.3	0.005%	0.000%
**Scenario 3: moderate non-additivity and non-linearity**						
Logistic regression	0.41	0.77	1.45	98.3	0.52%	0.06%
CART	0.22	0.35	1.64	49.0	0.48%	0.05%
Random forests	0.40	0.76	1.43	177.0	0.48%	0.06%
Boosted CART	0.19	0.38	0.75	20.3	0.01%	0.000%

**Table 2:** Spearman Correlations of Estimated Propensity Score Weights by Estimation Method and True Propensity Score Model Scenario.

Scenario 1: additivity and linearity				
	LGR	CART	RFRST	BOOST
LGR	1			
CART	0.46	1		
RFRST	0.77	0.55	1	
BOOST	0.83	0.53	0.89	1

Scenario 2: mild non-additivity and non-linearity				
	LGR	CART	RFRST	BOOST
LGR	1			
CART	0.44	1		
RFRST	0.75	0.55	1	
BOOST	0.81	0.53	0.90	1

Scenario 3: moderate non additivity and non-linearity				
	LGR	CART	RFRST	BOOST
LGR	1			
CART	0.39	1		
RFRST	0.63	0.59	1	
BOOST	0.65	0.56	0.90	1

LGR: logistic regression.

CART: classification and regression trees.

RFRST: random forests.

BOOST: boosted CART.

### Bias

**Figure 1:**
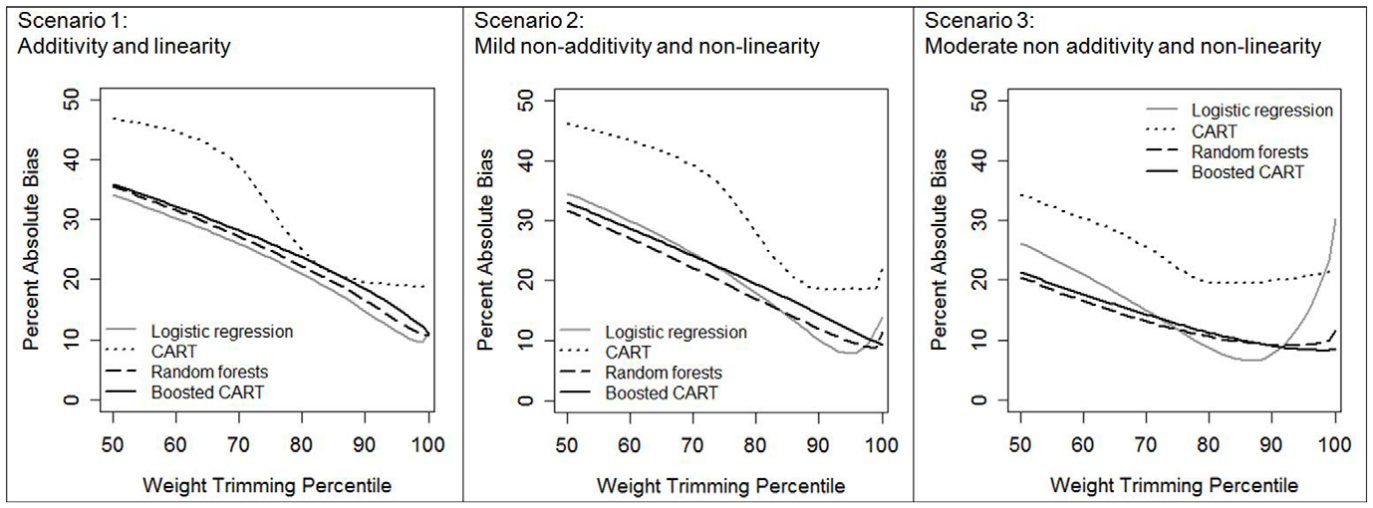
Average percent absolute bias in the estimate of treatment effect after propensity score weight trimming for 1000 simulated datasets of N = 500, by propensity score estimation method and degree of complexity in the true propensity score model scenario. Scenario 1: additivity and linearity; Scenario 2: mild non-additivity and non-linearity; Scenario 3: moderate non-additivity and non-linearity. The 100th percentile of weight trimming indicates no trimming was applied.

In the simplest scenario (1: additivity and linearity), all estimation methods except CART yielded little bias before trimming was applied (**Figure 1**). In part because of low bias without trimming, trimming in scenario 1 did not greatly reduce bias for any estimation method – in fact, trimming only increased bias for boosted CART, random forests, and CART. However, with increasing scenario complexity, the benefit of trimming became more apparent, particularly in the case of logistic regression and CART, where some (but not too much) trimming reduced bias in the estimated treatment effect. The optimal trimming level for logistic regression was at the 95th percentile in scenario 2 (mild non-additivity and non-linearity, 7.8% absolute bias versus 17.7% untrimmed), and at the 87th percentile in scenario 3 (moderate non-additivity and non-linearity, 6.5% absolute bias versus 30.3% untrimmed). In contrast, random forests benefited only slightly from trimming. For example, in scenario 3, even at the optimal trimming level of the 92nd percentile, the absolute bias was 9.0% trimmed versus 11.6% untrimmed. Boosted CART did not benefit at all from trimming in any scenario. The amount of trimming was also crucial: for all methods and scenarios, weight trimming beyond the optimal level substantially increased bias.

### Standard error

**Figure 2:**
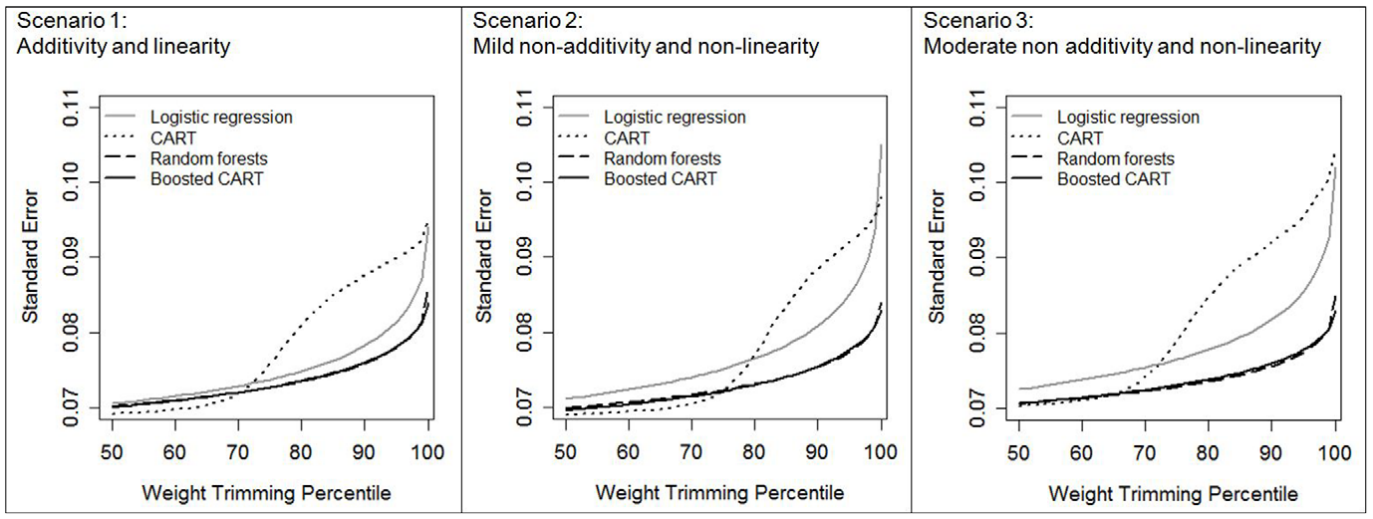
Average standard error in the estimate of treatment effect after propensity score weight trimming for 1000 simulated datasets of N = 500, by propensity score estimation method and degree of complexity in the true propensity score model scenario. Scenario 1: additivity and linearity; Scenario 2: mild non-additivity and non-linearity; Scenario 3: moderate non-additivity and non-linearity. The 100th percentile of weight trimming indicates no trimming was applied.

As expected, trimming decreased the standard error of effect estimates across all estimation methods and scenarios in a monotonic fashion (**Figure 2**). In particular, trimming sharply reduced the standard error for logistic regression (e.g., for scenario 3, at the 87th percentile, 0.080 versus 0.102 untrimmed). Although trimming reduced the standard errors for boosted CART and random forests, the reductions were not as dramatic, in part because the untrimmed standard errors for boosted CART and random forests (e.g., for scenario 3, 0.083 and 0.085 respectively) were already lower than for untrimmed logistic regression (0.102).

**Figure 3:**
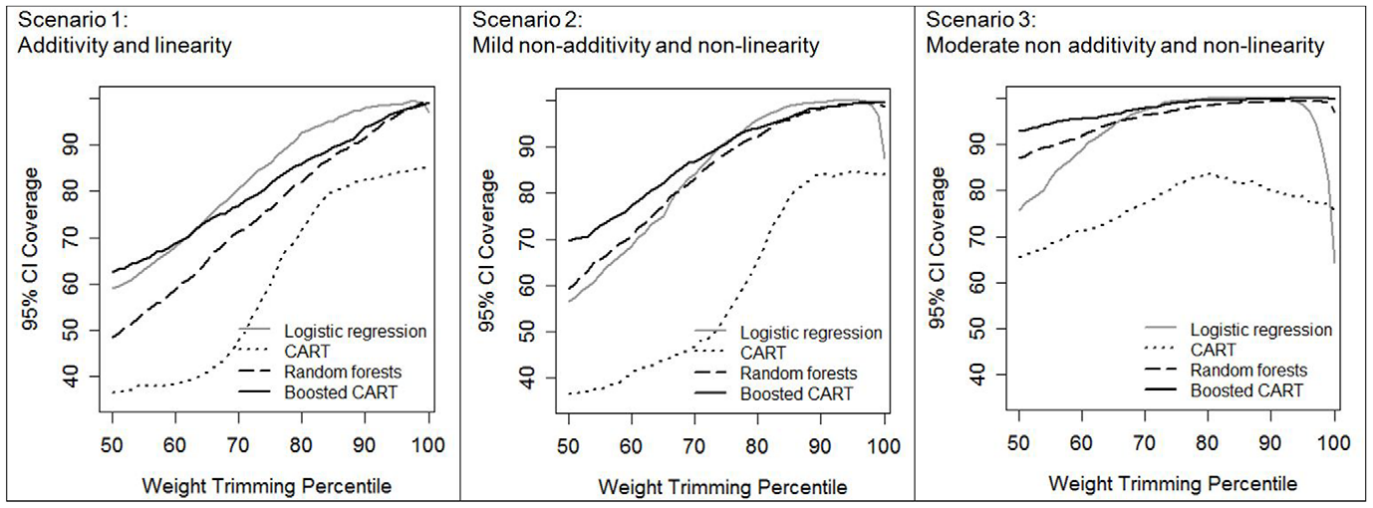
95% confidence interval coverage for 1000 simulated datasets of N = 500 after propensity score weight trimming, by propensity score estimation method and degree of complexity in the true propensity score model scenario. Scenario 1: additivity and linearity; Scenario 2: mild non-additivity and non-linearity; Scenario 3: moderate non-additivity and non-linearity. The 100th percentile of weight trimming indicates no trimming was applied.

*95% CI coverage*: In scenario 1, trimming only slightly improved CI coverage for logistic regression (at the optimal trimming level of the 98th percentile, coverage was 99.4% trimmed versus 97.0% untrimmed) and did not improve coverage for any of the other estimation methods (**Figure 3**). In more complex scenarios, trimming greatly improved the CI coverage of logistic regression even as the standard error decreased. Optimal trimming levels and corresponding coverage rates were as follows: logistic regression - scenario 2: 99.9% trimmed at the 95th percentile versus 87.5% untrimmed, scenario 3: 100% trimmed at the 92nd percentile versus 64.3% untrimmed. Trimming only improved coverage for CART in scenario 3, from 75.7% coverage untrimmed to 83.6% trimmed at the 81st percentile. Overall, trimming did not greatly improve 95% CI coverage rates for boosted CART or random forests.

## Discussion

In various simulation scenarios, weight trimming had the potential to improve the performance of propensity score weights, in particular for logistic regression-estimated weights. However, trimming did not improve the performance of propensity score weights estimated by boosted CART and random forests; in such situations, trimming can actually induce bias. The performance of boosted CART and random forests without weight trimming was similar to the best possible performance obtained by logistic regression with trimming. For all methods and scenarios, as the level of trimming increased, the standard error of the effect estimate progressively decreased. Note that here we refer to standard error in the statistical sense of estimated uncertainty in the effect estimate, absolute error (i.e., bias) may in fact increase even in settings where the estimated standard error decreases. Of course, decreasing the standard error is only good if the desired confidence interval coverage is maintained. Our results indicate that an ideal level of trimming exists such that bias and CI coverage are optimized, although this ideal level of trimming varies with scenario. As with other simulation studies, our results may not be generalizable to all situations utilizing propensity score weights. However, the scenarios used are similar to those typical in pharmacoepidemiologic studies, including common exposure, moderate magnitude of the exposure effect, collinearity of covariates, ranges of variables, and coefficients based on claims data modeling of statin use [20].

The present results demonstrate the detrimental effects of using misspecified propensity score models. Correctly specified logistic regression models performed quite well with low bias while misspecified logistic regression models that were missing important interactions and non-linearities in pre-treatment covariates produced high bias. The poor performance of misspecified logistic regression propensity score weighting due to large weights has been reported in other situations [23], [31]. However, large weights in and of themselves may not always be problematic when the propensity score model is correctly specified. Even when the distributions of weights estimated by random forests and logistic regression in our simulations were comparable (both with a number of large weights), trimming substantially improved logistic regression but not random forests (which performed well without trimming). This suggests that extreme weights alone are not largely responsible for increased bias and standard errors. Rather, it is the systematic misspecification of propensity scores by logistic regression models with only main effects terms that induced problems.

Although weight trimming appears to improve the performance of logistic regression-estimated propensity score weights in a variety of scenarios and is computationally easy to carry out, questions remain regarding how to implement trimming. The use of ad hoc adjustment methods such as propensity score weight trimming may be considered an admission of the failure of the underlying statistical methods used to estimate the propensity score. Hence, before trimming is implemented, it may be useful to examine and modify other aspects of the propensity score estimation process, especially concerning specification of non-linearities and interactions, variable selection, and variable parameterization [32], [33]. Machine learning algorithms such as boosted CART and random forests may be helpful in these tasks [21]. In addition, the distributions of weights should be examined to determine if results are sensitive to the few most extreme weights. It should be noted that methods to address extreme weights can be implemented directly within (instead of in addition to) machine learning methods. For example, Ridgeway and McCaffrey describe how the boosted CART algorithm (which we implemented here) has the effect of reducing the risk of obtaining spurious probabilities near 0 and 1 that lead to extreme weights [23]. Finally, without guidance on the optimal level of trimming, there exists the dangerous potential for trimming being used to artificially achieve a desired result. Bayesian methods to perform weight pooling and weight smoothing may be useful to objectively optimize weights [16], [34] for propensity score adjustment, although this has not been explored.

In conclusion, our results show that weight trimming can help reduce bias and standard error associated with logistic regression-estimated propensity score weights. However, weight trimming is of little to no utility for boosted CART and random forests-estimated propensity score weights, possibly because those methods perform so well already. We suggest that analysts should focus attention on improving propensity score model specification and rely less on weight trimming to optimize propensity score weighting.

## Supporting Information

Text S1 The R code used to generate the simulation data is presented in the Supporting Information Text S1.
(PDF)
