# Supplementary material for: Weight Trimming and Propensity Score Weighting
Source: PLoS One. 2011 Mar 31;6(3):e18174. doi: 10.1371/journal.pone.0018174 (PMC3069059; doi:10.1371/journal.pone.0018174)
Supplement: Text S1 — The R code used to generate the simulation data is presented in the Supporting Information Text S1. (PDF) [file pone.0018174.s001.pdf]

```r
# SUPPORTING INFORMATION TEXT S1
#~~~~~~~~~~~~~~~~~~~~Overview~~~~~~~~~~~~~~~~~~~~~#
# Author: Brian Lee
# This script is used for dataset generation for
# the manuscript "Weight trimming and propensity score weighting"
# in PLoS ONE 2011.
# The study design is based on methods printed in
# Setoguchi et al., "Evaluating uses of data mining
# techniques in propensity score estimation: a
# simulation study." Pharmacoepi Drug Saf 2008.

#~~~~~~~~~~~~~~~~~~~~Functions~~~~~~~~~~~~~~~~~~~~#
# function: generate continuous random variable correlated to variable x by rho
# invoked by the "F.generate" function
# Parameters -
#    x - data vector
#    rho - correlation coefficient
# Returns -
#    a correlated data vector of the same length as x

    F.sample.cor <- function(x, rho) {
            y <- (rho * (x - mean(x)))/sqrt(var(x)) + sqrt(1 - rho^2) * rnorm(length(x))
            #cat("Sample corr = ", cor(x, y), "\n")
            return(y)
    }

# function: generate simulation datasets
# inputs: sample size N, scenario
# outputs: 1 dataset of size N
    # binary variables: w1, w3, w5, w6, w8, w9
    # continous variables: w2, w4, w7, w10
    # confounders: w1, w2, w3, w4
    # exposure predictors only: w5, w6, w7
    # outcome predictors only: w8, w9, w10
    # correlations: (w1,w5)=0.2, (w2,w6)=0.9, (w3,w8)=0.2, (w4,w9)=0.9

    F.generate <- function(size, scenario) {
        w1 <- rnorm(size, mean=0, sd=1)
        w2 <- rnorm(size, mean=0, sd=1)
        w3 <- rnorm(size, mean=0, sd=1)
        w4 <- rnorm(size, mean=0, sd=1)
        w5 <- F.sample.cor(w1, 0.2)
        w6 <- F.sample.cor(w2, 0.9)
        w7 <- rnorm(size, mean=0, sd=1)
        w8 <- F.sample.cor(w3, 0.2)
        w9 <- F.sample.cor(w4, 0.9)
        w10 <- rnorm(size, mean=0, sd=1)

     #~~ dichotomize variables (will attenuate correlations above)
        w1 <- ifelse(w1 > mean(w1), 1, 0)
        w3 <- ifelse(w3 > mean(w3), 1, 0)
        w5 <- ifelse(w5 > mean(w5), 1, 0)
        w6 <- ifelse(w6 > mean(w6), 1, 0)
        w8 <- ifelse(w8 > mean(w8), 1, 0)
        w9 <- ifelse(w9 > mean(w9), 1, 0)

     #~~ scenarios for data generation models
        # A: model with additivity and linearity
        # B: mild non-linearity
        # C: moderate non-linearity
        # D: mild non-additivity
        # E: mild non-additivity and non-linearity
        # F: moderate non-additivity
        # G: moderate non-additivity and non-linearity

    # binary exposure modeling
        if (scenario == "A") {
            z.a_trueps <- (1 + exp( -(0 + b1*w1 + b2*w2 + b3*w3 + b4*w4 + b5*w5 + b6*w6 + b7*w7) )
            } else
        if (scenario == "B") {
```

```
            z.a_trueps <- (1 + exp( -(0 + b1*w1 + b2*w2 + b3*w3 + b4*w4 + b5*w5 + b6*w6 + b7*w7
                + b2*w2*w2) ) )^-1
            } else
        if (scenario == "C") {
            z.a_trueps <- (1 + exp( -(0 + b1*w1 + b2*w2 + b3*w3 + b4*w4 + b5*w5 + b6*w6 + b7*w7
                + b2*w2*w2 +b4*w4*w4 + b7*w7*w7) ) )^-1
            } else
        if (scenario == "D") {
            z.a_trueps <- (1 + exp( -(0 + b1*w1 + b2*w2 + b3*w3 + b4*w4 + b5*w5 + b6*w6 + b7*w7
                + b1*0.5*w1*w3 + b2*0.7*w2*w4 + b4*0.5*w4*w5 + b5*0.5*w5*w6) ) )^-1
            } else
        if (scenario == "E") {
            z.a_trueps <- (1 + exp( -(0 + b1*w1 + b2*w2 + b3*w3 + b4*w4 + b5*w5 + b6*w6 + b7*w7
                + b2*w2*w2 + b1*0.5*w1*w3 + b2*0.7*w2*w4 + b4*0.5*w4*w5 + b5*0.5*w5*w6) ) )^-1
            } else
        if (scenario == "F") {
            z.a_trueps <- (1 + exp( -(0 + b1*w1 + b2*w2 + b3*w3 + b4*w4 + b5*w5 + b6*w6 + b7*w7
                + b1*0.5*w1*w3 + b2*0.7*w2*w4 + b3*0.5*w3*w5 + b4*0.7*w4*w6 + b5*0.5*w5*w7
                + b1*0.5*w1*w6 + b2*0.7*w2*w3 + b3*0.5*w3*w4 + b4*0.5*w4*w5 + b5*0.5*w5*w6) ) )^-1
            } else
         {
         # scenario G
            z.a_trueps <- (1 + exp( -(0 + b1*w1 + b2*w2 + b3*w3 + b4*w4 + b5*w5 + b6*w6 + b7*w7
                + b2*w2*w2 + b4*w4*w4 + b7*w7*w7 + b1*0.5*w1*w3 + b2*0.7*w2*w4 +b3*0.5*w3*w5
                + b4*0.7*w4*w6 + b5*0.5*w5*w7 + b1*0.5*w1*w6 + b2*0.7*w2*w3 + b3*0.5*w3*w4
                + b4*0.5*w4*w5 + b5*0.5*w5*w6) ) )^-1
            }

        # probability of exposure: random number betw 0 and 1
        # if estimated true ps > prob.exposure, than received exposure (z.a=1)
            prob.exposure <- runif(size)
            z.a <- ifelse(z.a_trueps > prob.exposure, 1, 0)

        # continuous outcome modeling
            y.a <- a0 + a1*w1 + a2*w2 + a3*w3 + a4*w4 +a5*w8 + a6*w9 + a7*w10 + g1*z.a

        # create simulation dataset
            sim <- as.data.frame(cbind(w1, w2, w3 ,w4, w5, w6, w7, w8, w9, w10, z.a, y.a))
            return(sim)
    }

#~~~~~~~~~~~~~~~Global Variables~~~~~~~~~~~~~~~~~~~~#

    #~~ coefficients for data generation models
        b0 <- 0
        b1 <- 0.8
        b2 <- -0.25
        b3 <- 0.6
        b4 <- -0.4
        b5 <- -0.8
        b6 <- -0.5
        b7 <- 0.7
        a0 <- -3.85
        a1 <- 0.3
        a2 <- -0.36
        a3 <- -0.73
        a4 <- -0.2
        a5 <- 0.71
        a6 <- -0.19
        a7 <- 0.26
        g1 <- -0.4 # effect of exposure

#~~~~~~~~~~~~~~~~~~~~Calls~~~~~~~~~~~~~~~~~~~~#

    # this generates datasets
    # Example: Generate 1000 datasets of N=500 in scenario G
    simdata <- replicate(1000, F.generate(500, "G"))
```
